# First report on human-biting *Culex pipiens* in Sweden

**DOI:** 10.1186/s13071-016-1925-3

**Published:** 2016-12-07

**Authors:** Jenny C. Hesson, Martina Schäfer, Jan O. Lundström

**Affiliations:** 1 Department of Medical Biochemistry and Microbiology (IMBIM), Zoonotic Science Center, Uppsala University, Uppsala, Sweden; 2 Department of Epidemiology and Population Health, Institute of Infection and Global Health, University of Liverpool, Liverpool, UK; 3 Swedish Biological Mosquito Control Project, Nedre Dalälvens Utvecklings AB, Gysinge, Sweden

**Keywords:** Vector-borne infections, West Nile virus, Zoonoses, Mosquito vector, Arbovirus, Sindbis virus

## Abstract

*Culex* mosquitoes are vectors of several bird-hosted arboviruses that cause outbreaks in Europe, such as Sindbis virus and West Nile virus. Recently, the human-biting form of *Culex pipiens*, *Cx. pipiens* biotype *molestus*, was found causing big nuisance in a housing cooperative in Gothenburg in southern Sweden, confirmed by molecular identification. This is the first report of human-biting *Culex* in Scandinavia, signalling increased risk of arbovirus infection in northern Europe.

## Letter to the Editor

Mosquitoes of the genus *Culex* are the vectors of bird-hosted arboviruses that occasionally cause disease in humans, such as Sindbis virus (SINV) (Alphavirus) West Nile virus (WNV) and Usutu virus (USUV) (Flavivirus), all circulating in Europe. Seven potential vector species are recognised within the genus *Culex* in Europe, most of which show a preference for biting birds (ornithophilic). One of the species, the northern house mosquito *Cx. pipiens*, has been described as having two separate biotypes: *Cx. pipiens* biotype *pipiens* and *Cx. pipiens* biotype *molestus*, of which the latter fulfils certain criteria that separates it from the other: it does not hibernate in winter (homodynamic), it utilizes underground larval habitat (hypogeous), it can mate in confined spaces (stenogamous), it can lay its first egg batch without a blood meal (autogenous), and it feeds on humans (mammophilic). *Culex pipiens* biotype *molestus* has been reported from many central and southern European countries but never from Scandinavia [1–4]. Hybridisation of the two biotypes is quite common in the Mediterranean area, while hybrids are less commonly found in central Europe [3, 4]. *Culex pipiens* biotype *molestus* and potential hybrids are of high concern since they have the potential to serve as bridge vectors of these arboviruses from birds to humans, as they possess the vector competence of *Cx. pipiens* biotype *pipiens* [5] but an opportunistic feeding behaviour [6].

The last countrywide mosquito survey recognised three *Culex* species as endemic to Sweden: the ornithophilic species *Cx. torrentium*, *Cx. pipiens* biotype *pipiens* and *Cx. territans* that primarily feeds on ectothermic hosts [7]. Recently, an encounter with *Cx. modestus* that commonly bite man in central European countries has also been reported from southern Sweden [8].

In August 2016, one of the authors visited a private housing cooperative situated approximately 4 km south west of central Gothenburg, Sweden, that had experienced problems with mosquitoes biting tenants indoors. The housing cooperative is a property of approximately 4.5 ha with seven blocks of three-storey buildings. It is located in a residential area with similar buildings, intermixed with smaller park areas with deciduous bushes and trees. An online survey among the tenants showed that nearly 100 households (roughly a third of all flats in the cooperative), distributed throughout all the buildings and on all three floors of the cooperative, had problems with mosquitoes biting indoors. The earliest complaint of indoor biting was in winter/spring 2015, and since then the problem seems to have gradually increased, showing peaks in summer and autumn but also occurring during wintertime. The mosquitoes were reported to enter through open windows and balcony doors, as well as through the ventilation system on all three floors, and to be aggressive biters on humans during evening and night-time. Eleven female *Culex* mosquitoes were caught as they tried to bite humans, and *Culex* larvae were sampled in a catch basin just outside one of the affected buildings.

**Fig. 1:**
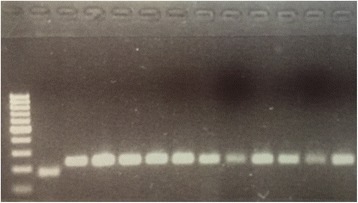
Electrophoresis gel confirming the presence of *Cx. pipiens* biotype *molestus* in Sweden. PCR products from a diagnostic assay separating *Cx. pipiens* biotype *pipiens* and *Cx. pipiens* biotype *molestus*, from left to right: a 100 bp size marker, 1 *Cx. pipiens* biotype *pipiens* (control), 11 *Cx. pipiens* biotype *molestus* collected in Gothenburg

The eleven adult *Culex* females caught indoors and ten larvae from the outside catch basin were used for DNA extraction and molecular species identification. Since *Cx. torrentium* is the most common *Culex* species in Sweden, a first assay for separating *Cx. torrentium* and *Cx. pipiens* (*s.l*.) was run [9], identifying all 11 adults and ten larvae as *Cx. pipiens* (*s.l*.). The DNA was then amplified in a second PCR designed to distinguish between *Cx. pipiens* biotype *pipiens* and *Cx. pipiens* biotype *molestus* [10], resulting in identification of all 11 adults as *Cx. pipiens* biotype *molestus* (Fig. 1), and the ten larvae as *Cx. pipiens* biotype *pipiens*.

Thus, environmental characteristics (homodynamic and mammalophilic), as well as molecular identification confirm the first report of presence of *Cx. pipiens* biotype *molestus* in Sweden. As of yet, however, no larval habitat has been identified.

This is the first time that a species of *Culex* has been reported to cause nuisance in Scandinavia. The most common *Culex* species in Sweden, *Cx. torrentium*, is often infected with SINV and is also a very efficient enzootic vector of the virus among birds [11, 12]. For transmission to humans to occur, it is also necessary to have a competent bridge vector that feeds opportunistically on both birds and humans. The discovery of *Cx. pipiens* biotype *molestus* in a densely populated area Sweden is important, as the presence of a bridge vector may increase the transmission risk of endemic SINV, as well as the risk for introduction and establishment of WNV and USUV. In addition to its vector potential, *Cx. pipiens* biotype *molestus* causes severe nuisance to humans, biting usually during night-time.

The origin of the Swedish *Cx. pipiens* biotype *molestus* is unknown. Population genetics studies have shown that populations of *Cx. pipiens* biotype *molestus* from different European countries are more closely related to each other than to nearby *Cx. pipiens* biotype *pipiens* populations [1, 2, 4, 6]. Thus *Cx. pipiens* biotype *molestus* is considered by most authors to be introduced into an area, rather than evolving through an adaptation of local *Cx. pipiens* biotype *pipiens* populations. The ways of introduction of *Cx. pipiens* biotype *molestus* to Sweden, and its distribution, will be further investigated. These are important steps towards a realistic risk assessment for mosquito-borne virus transmission to humans in Scandinavia.
